# Occult Papillary Thyroid Carcinoma Metastasis to the Sacrum and the Skull: An Unusual Presentation

**DOI:** 10.1155/2014/516549

**Published:** 2014-12-08

**Authors:** Fatima S. Jouhar, Asif Quadri, Bachar Afandi, Sadir Al Rawi

**Affiliations:** Department of Surgery, Division of Surgical Oncology, Tawam Hospital, Al Ain, Abu Dhabi, UAE

## Abstract

This case represents occult follicular variant of papillary thyroid carcinoma (FVPTC) with large metastasis to the sacrum. The patient, a 42-year-old female, presented after hemithyroidectomy for benign follicular adenoma with lower back pain associated with fever and sweating. A lytic lesion of the left sacral bone was found on the CT with biopsy showing metastatic carcinoma with morphology and immunophenotype of thyroid gland primary tumor proven to be FVPTC. The patient had completion thyroidectomy with benign pathology.

## 1. Introduction

Occult presentation of primary thyroid malignancy can be defined by the McGraw-Hill Concise Dictionary of Modern Medicine (2002) as “Unknown primary malignancy that is symptomless, which first manifest itself as metastasis or secondary-para-neoplastic phenomena” [1]. It can be classified into four categories: (1) thyroid carcinoma or microcarcinoma as an incidental finding after total thyroidectomy for benign mass, (2) radiological incidental finding of thyroid carcinoma with positive FNA result, (3) apparent thyroid metastasis with primary tumor unidentified prior to final histological specimen, and (4) symptomatic ectopic thyroid tissue with apparent metastasis [1]. In this case the patient presented with an apparent metastasis with pathology showing benign follicular adenoma.

## 2. Case Report

**Figure 1:**
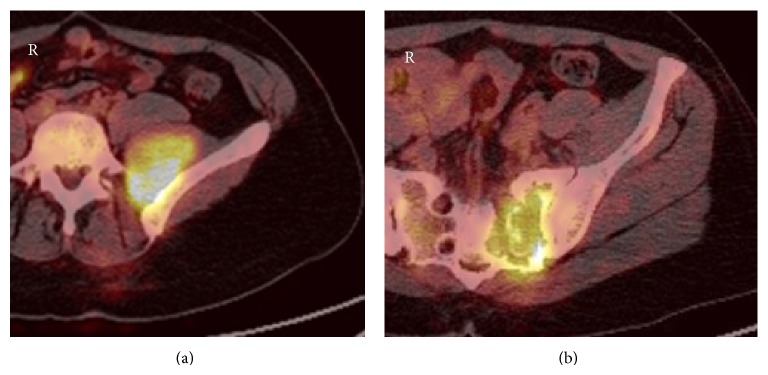
PET CT left osteolytic sacral lesion of 6.2 × 3.5 × 8.6 cm S1, S2, and S3 vertebral involvement extending to the neural foramina with very intense hypermetabolism.

**Figure 2:**
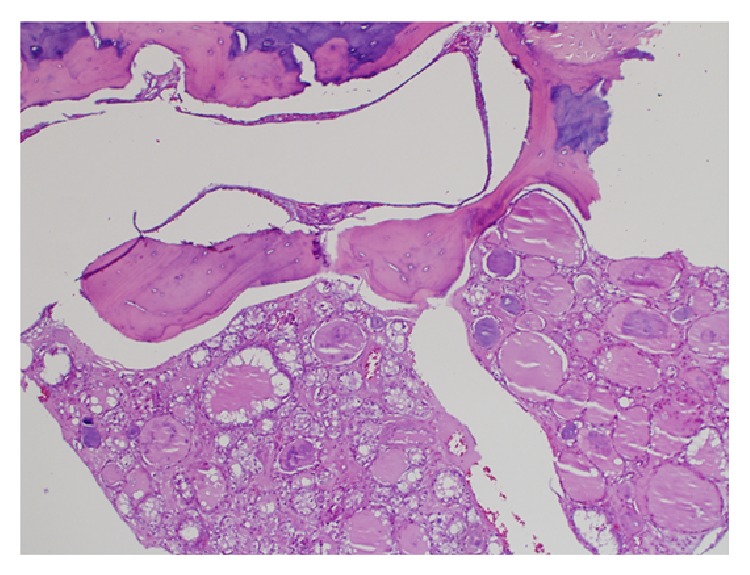
HE ×4 showing tumor invading bone.

A 42-year-old female presented with right neck swelling which was initially diagnosed as follicular adenoma. Patient underwent a right hemithyroidectomy and isthmus resection and was maintained on levothyroxine. Nine years later she presented with lower back pain associated with fever and sweating. CT scan of abdomen and pelvis showed lytic lesion of the sacrum. Three-phase bone scintigraphy showed substantial perfusion to the soft tissue mass located in left side of sacrum with photogenic area in the left sacroiliac joint region, surrounded medially with actively metabolic bony lesion. Sacral biopsy showed metastatic carcinoma with morphology and immunophenotype suggestive of thyroid gland primary tumor (positive thyroglobulin) and positive CK7+/CK20− (Figure 2). Positron emission tomography (PET)/CT scan showed large destructive left sacral lesion of 6.2 × 3.5 × 8.6 cm in size with soft tissue component involving S1, S2, and S3 vertebra, extending to the neural foramina of the involved vertebra and L5 vertebra, as well with very intense hypermetabolism (Figure 1). Patient was having decreased sense of vibration on her left lower limb. Patient was referred to the oncology center for palliative radiotherapy and completed 13 cycles. Patient was found to have left lobe thyroid nodule of 0.43 × 0.87 × 0.8 cm; FNA showed benign follicular hyperplastic nodule. Thyroglobulin level was elevated up to >600.0. Patient had completion thyroidectomy and lymph nodes dissection. Histopathology showed benign nodular hyperplasia of thyroid with negative lymph nodes. Iodine 131 uptake scan showed minimal uptake in thyroid bed, high uptake in pelvic region on the sacral mass, and hot round area in the posterior parietal region of the skull.

## 3. Discussion

**Figure 3:**
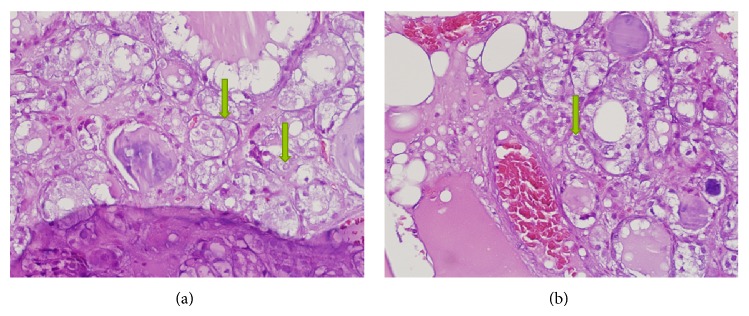
(a) HE ×20-nuclear overlapping is obvious in the lining tumor cells. (b) HE ×40-nuclear clearing in some nuclei (arrows).

This patient was found to have sacral mass which was diagnosed as an invasive tumor of thyroid origin. This was proven via the characteristic of the tumor with (1) bone invasion (Figure 2), (2) follicles filled with colloid, and (3) positive thyroglobulin and CK7+/CK20−. This current histopathology finding could be presented in papillary thyroid carcinoma, follicular thyroid carcinoma, and FVPTC, but the histopathology result was more suggestive of FVPTC which is represented in the higher magnification micrograph showing optical clear, overlapping, and grooved nuclei. This is highly suggestive of the papillary characteristics [2] (Figures 3(a) and 3(b)).

FVPTC has follicular architectural pattern with nuclear features of PTC. FVPTC can be misdiagnosed as follicular adenoma or follicular thyroid carcinoma in the presence of capsular or vascular invasion. It can present in larger size and in younger age groups. Although it is believed that FVPTC has similar prognosis of PTC, it can mimic the features of follicular neoplasm in terms of distal metastasis with absence of lymph node involvement and prevalence of vascular and capsular invasion. In spite of that, recent studies show FVPTC has favorable clinic pathological features compared to PTC, but similar long-term outcomes [3].

In this case the primary tumor was not found since both histopathology reports for the thyroid tissue in 2003 and 2013 revealed benign follicular adenoma. The absence of the primary tumor in this case can be either due to regression of tumor at the primary site with distant bone metastasis or due to a small primary tumor that could not be detected despite thorough examination [4].

It is believed that 1%–3% of thyroid cancer can develop distant metastasis. Occult clinical presentation delays the diagnosis and management of metastasis [5]. After the fourth decade 10% of patients with papillary thyroid cancer develop distant metastasis [6]. Metastases outside the neck and mediastinum are considered in 11% of patients with papillary thyroid carcinoma [5]. 2–13% of patients with well-differentiated thyroid cancer develop bone metastasis [7]. Bone is the second most commonly involved site after the lungs [6]. Compared to follicular thyroid carcinoma 28–97% papillary thyroid cancer has 1.4–7% of bone metastasis [7].

There have been cases in the literature reported in papillary thyroid metastasizing to the bone at different metastatic sites (Table 1). Bone metastasis increases mortality rate, decreases the quality of life, and shortens the patients' survival [8]. The presence of metachronous bone metastasis alone is a significant indicator of poor prognosis [9]. Large invasive bone metastases can be managed with aggressive surgical approach. Nevertheless, not all bone lesions are amenable to surgical excision [10].

Giving this case as an example, surgical excision in her condition will affect mobilization in addition to the neurological function due to the extensive invasionue to the extensive invasiveness to the bone and the involvement of the nerve roots at the level of the tumor. Alternative treatment such as arterial embolization or percutaneous radiofrequency ablation can be offered in her case [10]. Patient's case was discussed in the tumor multidisciplinary meeting and conservative palliative management was decided for the patient as surgery's risk exceeded the benefit in this case.

## 4. Conclusion

This case illustrates a rare presentation of metastatic lesion to the sacrum and the skull of histopathology highly suggestive of FVPTC. In addition to the rare finding of absence of primary lesion the histopathology result of the thyroidectomy showed benign follicular adenoma. Metastasis to the bone is considered more common in follicular thyroid carcinoma compared to papillary thyroid carcinoma which tends to increase the morbidity and mortality. Although FVPTC can mimic follicular thyroid cancer, it has similar prognosis to papillary thyroid carcinoma. Not all bone metastases can be managed surgically depending on the location and the postoperative outcome. Alternative treatment should be considered to improve patient morbidity.

## Figures and Tables

**Table 1:** Cases of occult and overt papillary thyroid carcinoma metastasis to the bone indicating different metastatic sites.

	Author	Age in years	Gender	Type	Metastatic site	Year
1	Sziklas et al. [11]	44	Male	Overt	Skull/ribs/pelvis	1985
2	Nishikawa et al. [4]	51	Male	Occult	Ribs	1998
3	Hashiba et al. [12]	74	Female	Overt	Skull	2006
4	Slim et al. [13]	67	Female	Overt	Malar bone	2012
5	Cardenas et al. [14]	59	Female	Overt	Skull/ribs/pelvis	2009
6	Cardenas et al. [14]	81	Male	Overt	Shoulder	2009
7	Chakravarthy et al. [15]	32	Female	Overt	Metacarpal bone	2010
8	Siddiq et al. [16]	59	Female	Overt	Iliac bone	2010
9	Özuğuz et al. [6]	35	Female	Overt	Ischium pubis	2011
10	Hugh et al. [17]	64	Female	Overt	Temporal bone	2011
11	Luna-Ortiz et al. [18]	30	Female	Overt	Sternum	2013
12	Nigam et al. [19]	48	Female	Overt	Skull	2012
13	Stojanie et al. [20]	56	Male	Overt	Sacrum	2012
14	Kutluhan et al. [21]	61	Male	Overt	Skull	2012
15	Luna-Ortiz et al. [18]	74	Female	Overt	Sternum/ribs	2013
16	Del Rio et al. [22]	60	Female	Overt	Pelvis	2013
17	Godbert et al. [23]	65	Male	Overt	Ribs	2013
18	Sachmechi et al. [24]	44	Female	Overt	Skull	2014
